# Metabolite Profiling and Anti-Aging Activity of Rice *Koji* Fermented with *Aspergillus oryzae* and *Aspergillus cristatus*: A Comparative Study

**DOI:** 10.3390/metabo11080524

**Published:** 2021-08-08

**Authors:** Hyunji Lee, Sunmin Lee, Seoyeon Kyung, Jeoungjin Ryu, Seunghyun Kang, Myeongsam Park, Choonghwan Lee

**Affiliations:** 1 Department of Bioscience and Biotechnology, Konkuk University, Seoul 05029, Korea; oasis7184@naver.com (H.L.); duly123@naver.com (S.L.); 2 COSMAX BTI R&I Center, Pangyo inno Valley E, 255 Pangyo-ro, Bundang-gu, Seongnam-si 13486, Korea; sykyung@cosmax.com (S.K.); jjyou@cosmax.com (J.R.); shyunk@cosmax.com (S.K.); msampark@cosmax.com (M.P.); 3 Research Institute for Bioactive-Metabolome Network, Konkuk University, Seoul 05029, Korea

**Keywords:** rice *koji*, microbe, solid-state fermentation, anti-aging effect, antioxidant activity

## Abstract

Rice *koji*, used as a starter for maximizing fermentation benefits, produces versatile end products depending on the inoculum microbes used. Here, we performed metabolite profiling to compare rice *koji* fermented with two important filamentous fungus, *Aspergillus oryzae* and *A. cristatus*, during 8 days. The multivariate analyses showed distinct patterns of primary and secondary metabolites in the two *koji*s. The rice koji fermented with *A. oryzae* (RAO) showed increased *α*-glucosidase activity and higher contents of sugar derivatives than the one fermented with *A. cristatus* (RAC). RAC showed enhanced *β*-glucosidase activity and increased contents of flavonoids and lysophospholipids, compared to RAO. Overall, at the final fermentation stage (8 days), the antioxidant activities and anti-aging effects were higher in RAC than in RAO, corresponding to the increased metabolites such as flavonoids and auroglaucin derivatives in RAC. This comparative metabolomic approach can be applied in production optimization and quality control analyses of *koji* products.

## 1. Introduction

Fermentation, which has a history of thousands of years, is increasingly being recognized as a method of enhancing nutrition and bioactivities of food products, in addition to processing and preserving them [1]. Rice *koji* is made by solid-state fermentation using steamed rice grains inoculated with microorganisms to secrete enzymes and produce beneficial metabolites. In recent years, various attempts to create delicate fermentation conditions have led to advanced fermentation efficacy and better food palatability [2,3]. Due to its advantages rice *koji* finds applications in industrial fields such as fermented foods and beverages and cosmetics [4,5,6].

Reactive oxygen species (ROS) are generated under conditions of oxidative stress and are by-products of aerobic metabolism. These free radicals can induce the degradation of biomolecules, resulting in oxidative damages, such as inflammation and acceleration of the skin aging process [7]. To develop a balance between ROS production and elimination, ROS scavengers, known as antioxidants, play an important role in alleviating oxidative stress and are mainly obtained from natural sources [8]. These free radicals are involved in the aging process, and scavenging them through the intake of antioxidants from natural sources is crucial in delaying aging [9]. In recent years, many studies have reported that rice *koji* can enhance the potential antioxidant activities of raw materials by improvement of the fermentation substrate [10,11].

The skin’s extracellular matrix (ECM) consists of collagen and elastin fibers, which promote elasticity of the skin to restore and maintain its original shape and state [12]. The destruction of the dermal ECM is an indicator of aging. It occurs due to the upregulation of collagen-degrading matrix metalloproteinase-1 (MMP-1), also known as collagenase. Therefore, studies of various phytochemicals that can slow down the skin aging process by stimulating collagen and elastin synthesis and inhibiting MMP-1 are increasing [13,14,15,16]. Seo et al. showed that fermented rice bran affects skin fibroblast collagen, inflammatory factor (IL-a), and MMP-1 [17]. Hence, various compounds found in rice, such as flavonoids and phenolic acids have antioxidant activity, and fermented rice *koji* has the potential to ameliorate the skin photoaging by UV radiation [18].

*Aspergillus,* a filamentous fungus, is a typical inoculum microbe for producing many beneficial metabolites such as simple sugars, fatty acids, and amino acids from *koji* in Asia. In particular, *Aspergillus oryzae* is the most common microorganism used in the production of *koji* because of its ensured safety and various enzymes, such as amylase, protease, and peptidase [19]. *Aspergillus cristatus* is used in tea fermentation, such as Fuzhuan brick tea, which has probiotics and protects against UVB-induced photoaging [20,21]. It has also been reported to enhance the antioxidant activity of various other raw materials [22,23]. Currently, efforts are increasingly being dedicated to improve the quality of fermentation starters [4,24]. Previous study have shown a comparative metabolic study of *Aspergillus* and *Bacillus*, widely used in rice *koji* [25]. However, there is a scarcity of information about the metabolomic differences between the same genera but different species of fungi. To select optimal microbes that can be introduced in the market for health with nutraceutical and cosmeceutical applications, there is a need for a comprehensive understanding of the metabolism of different inoculum microbes by comparing their bioactivity and metabolites.

In this study, we profiled the metabolites of rice *koji* fermented with different *Aspergillus spp*. (*A. cristatus* and *A. oryzae*) in terms of metabolomics to compare the metabolism of the two filamentous fungi. We also measured enzyme activity, antioxidant activity, and RNA expression of skin anti-aging factors (collagen, elastin, and MMP-1) to compare the two *koji*. Furthermore, we conducted correlation analysis to suggest potential candidate metabolites that contribute to antioxidant activity and skin anti-aging effects. A comprehensive analysis of MS-based metabolite profiling for comparing the two *koji* inoculums established a relationship between enzyme activities, metabolomes, and bioactivities. Here, we present a blueprint of the overall metabolic state, correlated with the bioactivities of the two different *koji* inoculums.

## 2. Results

### 2.1. Metabolic Profiling for Rice Koji Fermented with Different Aspergillus spp.

**Figure 1:**
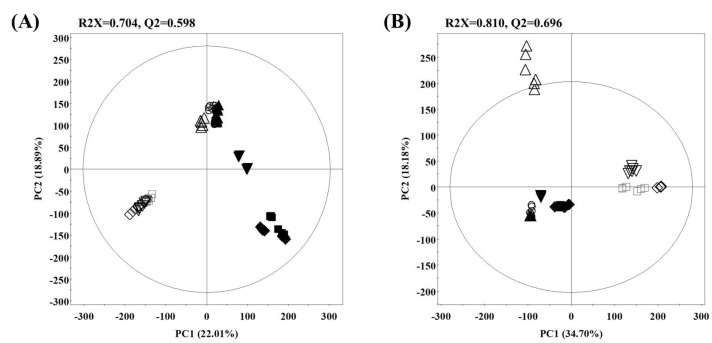
Principal component analysis (PCA) score plot from the (**A**) UHPLC–LTQ–Orbitrap–MS/MS and (**B**) GC–TOF–MS data sets of rice *koji* fermented with *Aspergillus cristatus* or *A. oryzae*. (filled symbols, *A. cristatus*; unfilled symbols, *A. oryzae*; ●, ○, 0 day; ▲, △, 2 day; ▼, ▽, 4 day; ■, □, 6 day; ◆, ◇, 8 day).

Different metabolomes of rice *koji* samples inoculated with *A. cristatus* or *A. oryzae* were compared using multivariate analysis according to the GC–MS and LC–MS data set. The principal component analysis (PCA) score plot obtained from the UHPLC–LTQ–Orbitrap-MS/MS and GC–TOF–MS revealed a total variance of 40.9% (PC1, 22.01%; PC2, 18.89%) and 52.88% (PC1, 34.70%; PC2, 18.18%), respectively (Figure 1A,B). Both PCA results indicated that the starting point of the fermentation was assembled, but consequently distinguished by different inoculation fungi according to different fermentation times. Partial least squares discriminant analysis (PLS-DA) elucidated statistical patterns same to the metabolite distribution in PCA (Supplementary Figure S1A,B).

As shown in the PCA obtained from the UHPLC–LTQ–Orbitrap–MS/MS analyses (Figure 1A), there are significant differences in the eighth day, and both eight day samples were subjected to an orthogonal partial least square discriminant analysis (OPLS-DA), which showed a clear separation by OPLS component 1, accounting for 86.11% of the variance in data (Supplementary Figure S1C). The 31 metabolites were selected from UHPLC–LTQ–Orbitrap–MS/MS data, which is considered a major contributor to the discrepancy in eighth day rice *koji*s fermented with two different inoculum microbes based on their variable importance in projection values (VIP > 1.0) and *p*-values (*p* < 0.05) from OPLS-DA analysis (Supplementary Table S1). These metabolites included 2 carboxylic acids, 5 phenolic acids, 7 flavonoids, 2 long chain fatty acids, 11 lysophospholipids, and 4 hydroquinones. The metabolites were tentatively identified by comparing published literature (molecular weight, molecular formula, retention time, mass fragment patterns, and UV absorbances) and data from an in-house library.

In addition, 20 primary metabolites from the GC–TOF–MS data that are contributed to distinctions in rice *koji* (VIP > 1.0 and *p* < 0.05) were determined by GC–TOF–MS to contain four organic acids, twelve sugar derivatives, and four fatty acids (Supplementary Table S2). These metabolites were identified by using standard compounds for comparing mass fragment patterns and retention times, in addition to an in-house library.

#### 2.1.1. Temporal Metabolomes for Rice *Koji* with Different *Aspergillus* spp. Inoculation According to Fermentation Time

**Figure 2:**
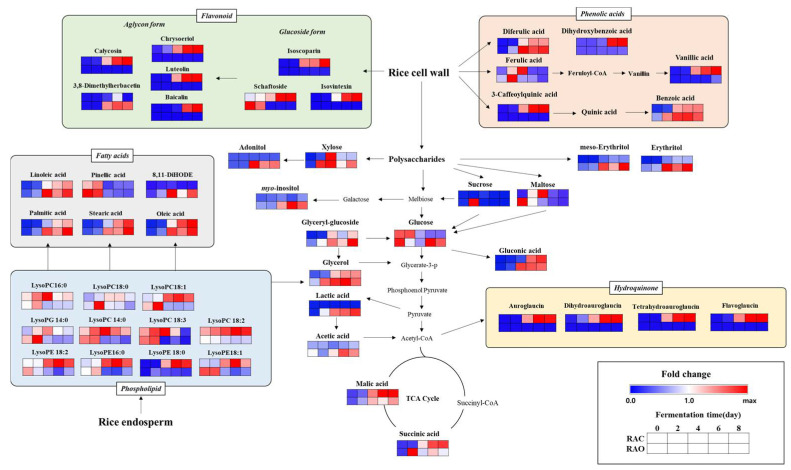
Scheme of the metabolic pathway and relative levels of metabolites in rice *koji* fermented with *Aspergillus cristatus* or *A. oryzae*. The pathway was adapted from the Kyoto Encyclopedia of Genes and Genomes (KEGG) database and modified. The colored squares represent the fold changes (blue to red) normalized by the average of all values for each metabolite.

The metabolic pathways of rice *koji* dependent on different inoculation microbes were represented by a heat map to visualize metabolite change patterns in accordance with fermentation times (Figure 2). The color on a blue-to-red gradient represents the mean-normalized relative abundance of each metabolite under each experimental condition. The trends of most metabolites in rice *koji* fermented with *A. cristatus* (RAC) and *A. oryzae* (RAO) showed a gradually increasing pattern with fermentation time. The metabolites associated with carbohydrate metabolism mostly represented an increasing pattern except for glucose, xylose, sucrose, and maltose, which are sugars. In addition, phenolic acid, flavonoids, and hydroquinone contents were enhanced with fermentation time, except for ferulic acid. Among the fatty acids, most metabolites showed an increasing pattern, while pinellic acid showed a decrease. Lysophospholipids presented disparate patterns with different fermentation times and inoculation fungi.

#### 2.1.2. Relative Disparity in the Level of Discriminant Metabolites in Rice *Koji* Fermented by *A. cristatus* or *A. oryzae*

As shown in Figure 2, the contents of primary and secondary metabolites exhibited different patterns in accordance with different inoculation fungi. In the case of glucose, which is the center of carbohydrate metabolism, the patterns of *A. cristatus koji* showed a decrease, whereas *A. oryzae koji* showed decreasing patterns at the initial fermentation point but gradually increased until the final fermentation point. Furthermore, the sugar alcohols were higher in RAO than in RAC. Particularly, auroglaucin derivatives were enhanced significantly only in RAC because they are a unique pigment compound produced by *A. cristatus*. In addition, most flavonoids were increased significantly in RAC compared to RAO, except for 3,8-dimethylherbacetin. Among the phenolic acids, diferulic acid and benzoic acid were increased in both samples, but dihydroxybenzoic acid, caffeoquinic acid, and vanillic acid were increased only in RAC. Lysophospholipids increased in RAC, but a contrasting tendency was observed in RAO. Fatty acids showed greater patterns of increase in RAO than in RAC.

### 2.2. Comparison of Enzymatic Production and Bioactivity in Rice Koji Fermented with Different Microorganisms

**Figure 3:**
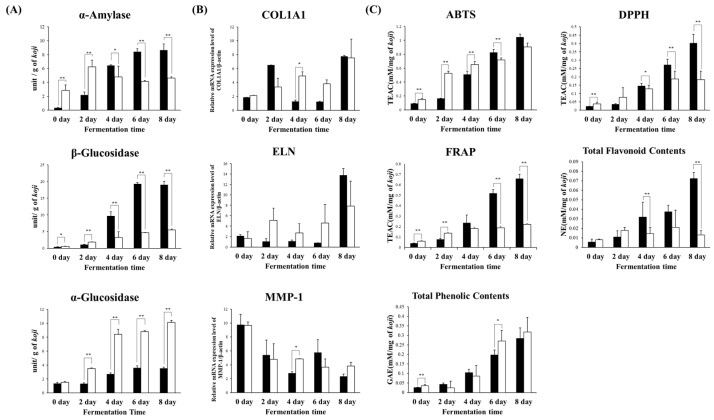
Comparison of enzyme production (**A**), skin anti-aging factor (**B**) and antioxidant activity, total flavonoid contents (TFC), total phenolic contents (TPC) (**C**) in rice *koji* fermented with different *Aspergillus spp*. (black color, *A. cristatus*; white color, *A. oryzae*). The enzymatic activities are α-amylase activity, *β*-glucosidase activity, and α-glucosidase activity (**A**). The relative mRNA expression level is measured for the following: collagen (COL1A1), elastin (ELN), and matrix metalloproteinase-1 (MMP-1) (**B**). The antioxidant activities depicted are ABTS, DPPH radical scavenging, FRAP, total flavonoid content, and total phenolic content (**C**). Significant differences between different inoculation microbes were identified by *t*-test (* *p* < 0.05, ** *p* < 0.01).

To compare the phenotypes of RAC and RAO, we evaluated the enzyme activity and anti-aging effects on skin cells, and antioxidant activity, total flavonoid contents (TFC) and total phenolic contents (TPC) (Figure 3). Enzyme production of both the *koji*s increased with fermentation time, except for *α-*amylase in RAO. Interestingly, the *α*-glucosidase content was twice higher in RAO than in RAC with 10.12 and 3.52 units respectively; in contrast, *β*-glucosidase content was forth higher in RAC than in RAO with 19.05 unit and 5.49 unit respectively, in accordance with fermentation times. The functional phenotype of both the *koji*s (antioxidant activity and skin anti-aging factor) indicated that the rice *koji* with *A. cristatus* had higher antioxidant activities in ABTS, DPPH, FRAP at the final fermentation time (8 days) with 1.05, 0.40, 0.66 TEAC (Trolox equivalent antioxidant capacity) respectively. Additionally, the content of flavonoid was higher in RAC than RAO with 0.07 NE (naringin equivalent) and 0.01 NE respectively. Whereas the content of total phenol was higher in RAO than RAC with 0.32 EGA (equivalent gallic acid) and 0.28 EGA respectively. The results of skin anti-aging factor (elastin, collagen, and MMP-1) indicated that at the termination of the fermentation, RAC had higher relative collagen and elastin RNA expression level with 7.77 and 13.76 and lower relative MMP-1 RNA expression level with 2.35 compared to *β*-actin. Meanwhile, RAO showed a gradual increase in RNA expression of elastin and collagen following fermentation.

**Figure 4:**
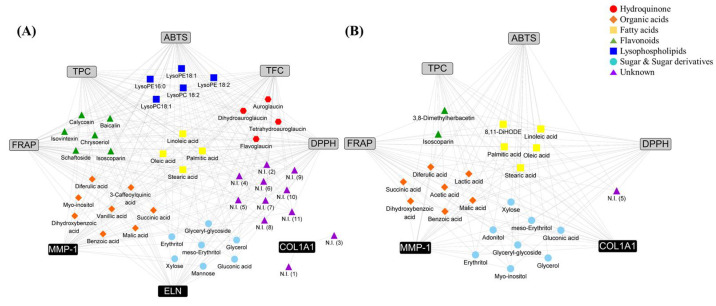
The metabolites that have a Pearson’s correlation coefficient value higher than 0.5 are represented by a network map in rice *koji* fermented with (**A**) *Aspergillus cristatus* or (**B**) *A. oryzae*. The box symbols represent bioactivities (gray color, antioxidant activity, TPC and TFC; black color, skin anti-aging effect on cell) and the colored symbols indicate the metabolites (same series were distinguished by different color and shape; ●, hydroquinone; ◆, organic acids; ■, fatty acids; ▲, flavonoids; ■, lysophospholipids; ●, sugar and sugar derivatives; ▲, unknown).

To determine the metabolites that potentially contributed to bioactivity, a correlation analysis between fermented *koji* metabolites and bioactivities was conducted (Supplementary Figure S2). Overall, the Pearson’s correlation coefficient map showed the RAC higher correlation with bioactivities than RAO. In RAC, organic acids, flavonoids, lysophospholipids, fatty acids, hydroquinone, sugar derivatives were showed high positive correlation with bioactivities. For RAO, organic acids, flavonoids and fatty acids, and sugar derivatives were indicated positive correlation with bioactivities. The metabolites that had a Pearson’s correlation coefficient value higher than 0.5 are represented in a network map (Figure 4). In both the *koji* products, organic acids, fatty acids, flavonoids, and sugar derivatives were potential contributors of bioactivities. RNA expression of elastin was associated with metabolites of RAC, whereas RNA expression of collagen was associated with metabolites of RAO. In addition, TFC showed a correlation with RAC. Furthermore, lysophospholipids and hydroquinone were strong antioxidant activity contributors to RAC.

## 3. Discussion

Different parts of rice, such as husk, bran, embryo, and endosperm, from the surface to the interior, have different chemical compositions [26]. In particular, rice bran contains various phenolic acids and flavonoids, which are known to exhibit antioxidant activity. In addition, the rice cell wall is composed of an arabinoxylan structure that includes xylose, arabinose, ferulic acid, and diferulic acid [27]. The rice cell wall is generally hard to penetrate, and rice *koji* offers the advantage of easy penetration of the rice cell wall by various enzymes such as protease and glucosidase from inoculum microbes [24]. Therefore, rice *koji* shows higher levels of tyrosinase inhibitory activity and antioxidant activities than its raw materials because it contains valuable enriched compounds [28].

We followed the metabolomics approach for rice *koji* fermented with two different filamentous fungi, which elucidated significant differences in enzyme activity, production of metabolites, and bioactivities. Activities of various enzymes such as *α*-amylase, *α*-glucosidase, and *β*-glucosidase produced by the inoculated *A. cristatus* and *A. oryzae* increased with fermentation time (Figure 3). Because these enzymes break down the arabinoxylan structure, diverse phenolic acids were separated from the rice cell wall in both the samples, as shown in Figure 2. These phenolic acids are potential antioxidants that alleviate oxidative stress [29]. Thus, antioxidant activities and TPC assay increased with increasing fermentation time as the phenolic acid content was enhanced (Figure 2 and Figure 4). In particular, RAC has a higher content of flavonoids than RAO because it has a higher level of *β*-glucosidase, which hydrolyzes the *β*-glycosidic linkage from the rice cell wall, during the growth of *A. cristatus* in rice *koji*. In addition to detaching from the rice cell wall, *β*-glucosidase hydrolyzes the flavonoid glucoside form to an aglycon form that possesses higher antioxidant activity [30]. The increased flavonoid glucoside form and aglycon form increases antioxidant activities such as ABTS, DPPH, FRAP, and TFC, which may affect the antioxidant activity of RAC, as shown in the correlation network map (Figure 4). This phenomenon was also observed in a previous study that showed biotransformation of glucoside isoflavones to aglycones and increasing patterns in antioxidant activity according to fermentation time in soybean fermented with *A. cristatus* [31].

RAO has a higher level of *α*-glucosidase activity that cleaves *α*-glycosidic linkages and generates higher content of glucose. Besides the fact that the glucose is the main carbon source for fungus, in RAC, the glucose level decreased following fermentation because it was used for the synthesis of secondary metabolites such as auroglaucin derivatives, which are distinctive pigment compounds produced by *A. cristatus,* and not *A. oryzae*. Previous studies have reported that auroglaucin derivatives have activity in DPPH and assumed as the potential antioxidant compounds [32]. Furthermore, the collapsed rice cell wall could allow enzymes to penetrate into the innermost parts of the rice [24]. Hence, more and more metabolites could be extracted freely without interruption from the rice outer wall.

In the correlation network map between bioactivities and metabolites of both RAO and RAC (Figure 4), the common tendencies were that flavonoids, organic acids, sugar derivatives, and fatty acids were suggested as potential contributors to bioactivities. Flavonoids and phenolic acids are renowned antioxidants and have many advantages with respect to various functions. Due to their ability to alleviate oxidative stress, they are used to enhance food quality and ameliorate skin aging [33]. Additionally, a previous study has reported that fatty acids and antioxidants could create a synergistic effect for the prevention and management of skin aging [34].

On the other hand, auroglaucin and lysophospholipid derivatives serve as additional contributors to metabolites in RAC [35]. The auroglaucin derivatives have antioxidant activities, as stated above, and therefore, we assume that they have the potential to terminate free radical chain reactions to relieve skin stresses. Yahagi et al. demonstrated that lysophospholipids could maintain skin moisturization by enhancing the expression of factors associated with the skin barrier and hydration functions in the skin [36]. Moisturization is a vital factor for healthy skin because dryness induces skin impairment that is characterized by roughness, scaly skin and fine wrinkles [37,38]. We estimate that auroglaucin and lysophospholipids have better skin anti-aging effects at the final fermentation stage in RAC than in RAO. Zhao et al. demonstrated that Fuzhuan brick tea, which contains the dominant fungus *A. cristatus*, can inhibit photoaging via quenching of ROS and triggering of Nrf2 signaling cascades [21]. Therefore, we assume that RAC offers a higher anti-aging potential than RAO by acting through indirect routes such as establishing better skin conditions for abundant moisture and relieving free radical stress.

Overall, we believe that the enhanced fatty acids, phenolic acids, flavonoids, lysophospholipids, and hydroquinones may increase antioxidant activities and improve RNA expression of elastin and collagen, as well as suppress RNA expression of MMP-1, at the end of fermentation. These compounds exhibited different patterns of change in metabolites according to the inoculum fungus and affected various bioactivities. This study elucidated the difference in overall metabolism between different species of the same Aspergillus genus by using a metabolomics approach. In addition, different enzyme activities influenced the production of different metabolites and induced different bioactivities in RAO and RAC.

## 4. Materials and Methods

### 4.1. Chemicals and Reagents

Analytical grade methanol, acetonitrile, and water were purchased from Fisher Scientific (Pittsburgh, PA, USA). Reagent grade chemicals, including methoxyamine hydrochloride, pyridine, and N-methyl-*N*-(trimethylsilyl)-trifluoroacetamide (MSTFA), were obtained from Sigma Chemical Co. (St. Louis, MO, USA).

Analytical-grade chemicals, namely, acetic acid, 2,2-azinobis(3-ethylbenzothiazoline- 6-sulfonic acid) diammonium salt (ABTS), Folin–Ciocalteu’s phenol, formaldehyde solution, formic acid, methoxyamine hydrochloride, p-nitrophenol, p-nitrophenol β-d-glucopyranoside (p-NPG), potassium persulfate, pyridine, sodium hydroxide, sodium acetate, starch, N-methyl-*N*-(trimethylsilyl)trifluoroacetamide (MSTFA), 6-hydroxy-2,5,7,8-tetramethylchroman-2-carboxylic acid (Trolox), and tyrosine were purchased from Sigma-Aldrich (St. Louis, MO, USA). Sodium carbonate, sodium dihydrogen phosphate, and disodium hydrogen phosphate were purchased from Junsei Chemical Co., Ltd. (Tokyo, Japan).

### 4.2. Sample Preparation and Extraction

The *koji* molds *A. oryzae* KCCM 11372 (Korean Culture Center of Microorganism, KCCM; Republic of Korea) and *A. cristatus* (*Aspergillus cristatus* Cosmax-GF from Cosmax BTI R&I center; Seongnam, Korea) were used for fermentation of rice and separately inoculated. Each microorganism was maintained on malt extract agar (malt extract, 20 g; glucose, 20 g; peptone, 1 g; agar, 20 g/L) at 28 °C. The bioprocess of fermentation steps for *koji* production was adapted from Lee et al. [11]. The rice *koji* samples fermented with *A. oryzae* and *A. cristatus* were harvested every 2 days (from day 0 to day 8) and stored at deep freezing conditions (−80 °C) until further analyses. All samples were prepared with two biological replicates.

The method of extraction of rice *koji* sample was adapted from Lee et al. with slight modifications [11]. Briefly, the pulverized freeze-dried rice *koji* samples (5 g) were extracted by adding 80% aqueous ethanol (40 mL) and agitating on an orbital shaker (200 rpm for 24 h) at room temperature. After centrifugation of the samples at 10,000 rpm for 5 min at 4 °C, the supernatants were filtered with a 0.22 μm Millex GP filter (Merck Millipore, Billerica, MA, USA). The filtered sample extracts were dried using a speed vacuum concentrator (Hanil, Seoul, Korea) and the dry weight was measured to evaluate the extraction yield.

### 4.3. GC–TOF–MS Analysis

The derivatization steps of extracted rice *koji* samples were as described by Lee et al. [11]. GC–TOF–MS analysis was conducted on an Agilent 7890A GC system (Santa Clara, CA, USA) with a Pegasus HT TOF-MS (Leco Corporation, St. Joseph, MI, USA). The carrier gas (helium) was used with an RTx-5MS (30 m length × 0.25 mm inner diameter, J&W Scientific, Folsom, CA, USA) at a constant flow rate of 1.5 mL/min. The temperatures of the injector and ion source were maintained at 250 and 230 °C, respectively. The oven temperature was maintained at 75 °C for 2 min and then increased to 300 °C at 15 °C/min, which was sustained for 3 min. Then, 1 μL of sample was injected with a mass scan range of m/z 50–800. All sample analyses were performed with three analytical replicates.

### 4.4. UHPLC–LTQ–Orbitrap–MS Analysis

The extracted rice *koji* samples were analyzed for secondary metabolites using ultrahigh-performance liquid chromatography linear trap quadrupole orbitrap tandem mass spectrometry (UHPLC–LTQ–Orbitrap–MS/MS) using the protocols described by Kwon et al. [39]. Each sample was separated using a Phenomenex KINETEX^®^ C18 column (100 mm 2.1 mm, 1.7 m particle size; Torrance, CA, USA). The mass spectra and photodiode array range in both positive and negative ion modes were tuned for m/z 100−1000 and 200−600 nm, respectively.

### 4.5. Data Processing and Statistical Analysis

The raw GC–TOF–MS and UHPLC–LTQ–Orbitrap–MS/MS data were transformed to netCDF (*.cdf) format using Leco ChromaTOF and Thermo Xcalibur software, respectively. The respective net CDF (*.cdf) files were subjected to MetAlign (http://www.metalign.nl (accessed on 13 July 2021)) software-mediated data processing using the protocols described by Lee et al. [11,24]. The mass spectrometric data, which represent the suitable peak mass (m/z), retention times (min), and peak area information as variables, were evaluated using SIMCA-P+ 12.0 software (Umetrics, Umea, Sweden) for multivariate statistical analysis. Before principal component analysis (PCA), partial least squares discriminant analysis (PLS-DA), and orthogonal partial least square discriminant analysis (OPLS-DA), the data sets were log-transformed, and unit variance was scaled to compare the rice *koji* fermented with different fungi. PASW Statistics 18 (SPSS, Inc., Chicago, IL, USA) was used to test for significant differences (*p*-value of < 0.05) by one-way analysis of variance and to calculate the correlation coefficient values for a correlation map. The correlation network map between metabolite that have a Pearson’s correlation coefficient value higher than 0.5 and bioactivities were constructed with the Cytoscape software (https://www.cytoscape.org/ (accessed on 13 July 2021)). The identification of tentative metabolites was carried out by matching the molecular weights and molecular composition, retention time, mass fragment patterns, and absorbance of ultraviolet (UV) data from the literature and our in-house library.

### 4.6. Determination of Enzymatic Activities

Enzymatic activity assays for *α*-amylase, *β*-glucosidase, and *α*-glucosidase were performed according to previous studies [25,40,41]. A 10 g quantity of each rice *koji* sample was extracted in 90 mL of water by shaking on an orbital shaker at 120 rpm and 25 °C for 1 h. After filtering the samples, the supernatants were used to evaluate enzyme activities.

### 4.7. Determination of Antioxidant Activities and Total Phenolic and Flavonoid Contents

To determine the antioxidant activity of the rice *koji* samples, ABTS, DPPH, ferric reducing antioxidant power (FRAP), total phenolic contents (TPC), and total flavonoid contents (TFC) assays were conducted in triplicate.

The ABTS and FRAP assays were performed using the method described by Lee et al. [24]. In brief, the ABTS stock solution diluted with distilled water to achieve a final absorbance of 0.7 ± 0.02 at 750 nm (180 μL) was added to each sample extract (20 μL) in a 96-well plate. The reaction was allowed to take place for 6 min in the dark at room temperature. The absorbance was measured at 750 nm using a spectrophotometer. For the FRAP assay, a mixture of 300 mM acetate buffer (pH 3.6), 20 mM iron (III) chloride, and 10 mM 2,4,6-tripyridyl-S-triazine (TPTZ) solution in 40 mM HCl (10:1:1, *v*/*v*/*v*) was prepared. The sample (10 μL) was mixed with 300 μL of FRAP reagent and incubated at room temperature for 6 min. The absorbance was measured at 570 nm. The DPPH assay was carried out following the method adapted from Won et al. [42], where 180 µL of the DPPH stock solution (0.2 mM in ethanol) was mixed with 20 µL of the rice *koji* with two different fungal extracts in 96-well plates and allowed to react for 20 min at room temperature in the dark. The free radical absorbance by DPPH was measured at 515 nm. The results of ABTS, FRAP, and DPPH are represented as the Trolox equivalent antioxidant capacity (TEAC) concentration (mM) per milligram of *koji*. The standard concentration curves ranged from 0.0078 mM to 1 mM TEAC.

For the TFC and TPC assays, a method used by Lee et al. [25] was followed. For the TFC assay, 20 µL of each rice *koji* sample was mixed with 20 µL of 1 N NaOH and 180 µL of 90% diethylene glycol in a 96-well plate. After incubation of the mixture for 60 min at room temperature, the absorbance was measured at 405 nm. TFC is presented as the naringin equivalent (NE) concentration (mM) per milligram of *koji*. The standard concentration curve was linear between 0.0027 and 0.3445 mM NE. For the analysis of the TPC assay, 20 µL of each sample was incubated with 100 µL of 0.2 N Folin–Ciocalteu reagent in 96-well plates at room temperature for 6 min. Then, 80 µL of 7.5% sodium carbonate (Na_2_CO_3_) solution was added to the mixture and allowed to react for 60 min at room temperature. Finally, the absorbance was evaluated at 750 nm. The results are indicated as gallic acid equivalent (GE) concentrations (mM) per milligram of *koji* in a standard concentration range of 0.0230–2.9391 mM GE.

### 4.8. Cell Cultures

Primary human dermal fibroblasts (HDFs), a related culture medium, and DetachKit were purchased from PromoCell (Heidelberg, Germany). The HDFs were cultured in specific fibroblast medium (Fibroblast Growth Medium 2, PromoCell, Cat no. C-23020) abundant with Supplement Mix/Fibroblast Growth Medium 2 (PromoCell, Cat no. C-39325) and 1% penicillin-streptomycin (PS) at 37 °C in a 5% CO_2_ incubator. When the cultured HDFs reached almost 80% confluence, they were sub-cultured or seeded into the proper wells for the different treatments and further assays.

### 4.9. Real-Time Polymerase Chain Reaction

To isolate and quantify the total RNA from the cell pellets, Trizol reagent was used, and the analysis was done using a spectrophotometer. The synthesis of cDNA was carried out in a total reaction volume of 20 µL; the reaction mixture consisted of 2 µg of total RNA, oligo (dT), and reverse transcription premix under the following reaction conditions: 45 °C for 45 min, followed by 95 °C for 5 min. RT-PCR was used for quantification of gene expression, and the results were subsequently analyzed using the StepOne PlusTM system software (Applied Biosystems, Foster City, CA, USA). RT-PCR amplifications were conducted using SYBR Green PCR Master Mix with premixed ROX (Applied Biosystems, Foster City, CA, USA) and primers (Bioneer, Daejeon, Korea) in an ABI 7300 instrument following the manufacturer’s protocol. The reaction conditions were as follows: initiation at 95 °C for 10 min, followed by cycling conditions of 95 °C for 15 s, 60 °C for 30 s, and 72 °C for 30 s for 40 cycles. *β*-actin was used as an internal control.

## 5. Conclusions

In conclusion, rice *koji* showed the production of different metabolites and bioactivities according to different *Aspergillus* species used. The higher levels of flavonoids and auroglaucin derivatives in RAC resulted in a higher antioxidant activity than in RAO. In addition, the synergistic effects of fatty acid and antioxidant compounds found in both the *koji* were associated with the RNA expression of the skin anti-aging factor. Auroglaucin derivatives and lysophospholipids found in RAC were also candidates that could be associated with RNA expression of skin anti-aging factors. Therefore, even though rice *koji* is fermented using members of the same genus (*Aspergillus*), there are significant differences in enzyme activities and metabolites for the different species, and they affect bioactivities such as antioxidant and anti-aging activities. Hence, this study provides comprehensive insight, as well as logic for a rational choice of inoculation microbes, with respect to metabolomics, to improve the quality of commercial production of *koji*.

## Data Availability

The data presented in this study are available on request from the corresponding author.

## Supplementary Materials

The following are available online at https://www.mdpi.com/article/10.3390/metabo11080524/s1, Figure S1: The PLS-DA score plot (A,B) and OPLS-DA score plot (C) for rice *koji* fermented with *Aspergillus cristatus* or *A. oryzae* were obtained from UHPLC–LTQ–Orbitrap-MS/MS (A,C) and GC–TOF–MS (B)., Table S1: List of significantly distinct metabolites from rice *koji* with different *Aspergillus spp*. during fermentation identified by UHPLC–LTQ–Orbitrap-MS/MS, Table S2: List of significantly distinct metabolites from rice *koji* with different *Aspergillus spp*. during fermentation identified by GC-TOF-MS, Figure S2: Correlation map of bioactivities (skin cell effect and antioxidant activity) and rice *koji* fermented with *Aspergillus cristatus* or *A. oryzae* metabolites according to Pearson’s correlation coefficient. Each square indicates Pearson’s correlation coefficient values (r).
