# Supplementary material for: Metabolite Profiling and Anti-Aging Activity of Rice Koji Fermented with Aspergillus oryzae and Aspergillus cristatus: A Comparative Study
Source: Metabolites. 2021 Aug 8;11(8):524. doi: 10.3390/metabo11080524 (PMC8398186; doi:10.3390/metabo11080524)
Supplement: Supplementary file 1 [file metabolites-11-00524-s001.zip › metabolites-1317995-supplementary.pdf]

metabolites

MDPI

Supplementary data

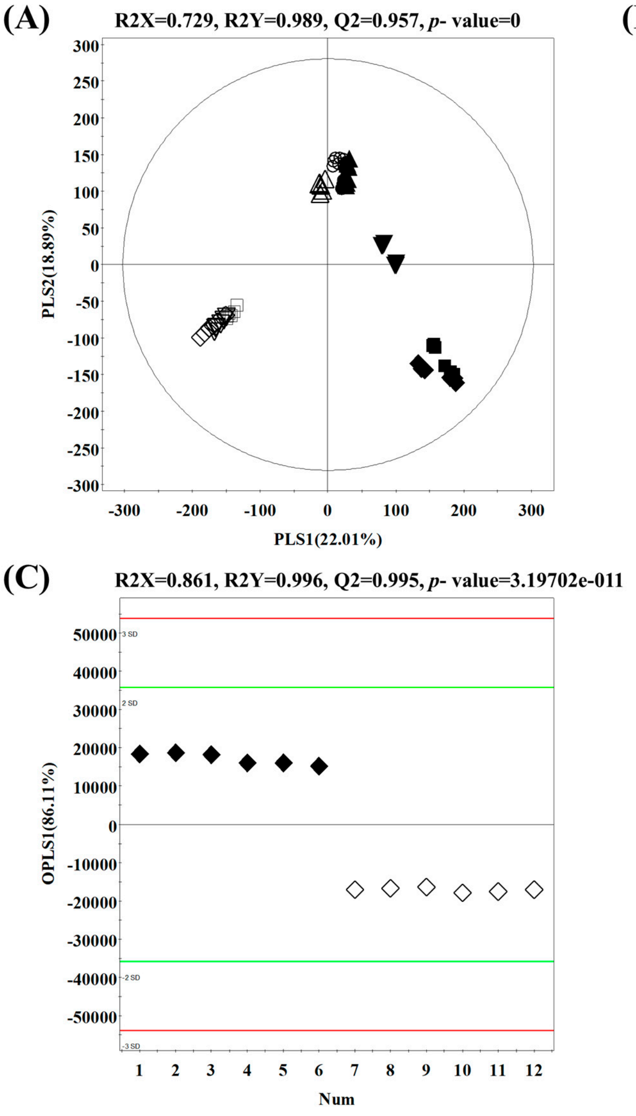

Figure S1. The PLS-DA score plot (**A**, **B**) and OPLS-DA score plot (**C**) for rice *koji* fermented with *Aspergillus cristatus* or *A. oryzae* were obtained from UHPLC-LTQ-Orbitrap-MS/MS (**A**, **C**) and GC-TOF-MS (**B**). (filled symbols, *A. cristatus*; unfilled symbols, *A. oryzae*; ●, ○, 0 day; ▲, △, 2 day; ▼, ▽, 4 day; ■, □, 6 day; ◆, ◇, 8 day)

Table S1. List of significantly distinct metabolites from rice *koji* with different *Aspergillus spp*. during fermentation identified by UHPLC-LTQ-Orbitrap-MS/MS.

No.	Metabolite	RT[a]	*m/z*		M.W.[b]	Molecular Formula	Δ ppm	MS[c] fragment pattern (*m/z*)	λmax	I.D.[d]
			$[M-H]^-$	$[M+H]^+$						
Carboxylic acids										
1	Malic acid	0.74	133.0152	157.0492	134	C4H6O5	6.491	(-)133>115>70	-	REF[e][1]
2	Succinic acid	0.95	117.0202	119.0895	118	C4H6O4	7.162	(-)117>99,73	-	REF[2]
Phenolic acid										
3	Dihydroxybenzoic acid	3.92	153.0201	177.0541[f]	154	C7H6O4	5.019	(-)153>109>81,65	257	REF[3]
4	Benzoic acid	4.04	121.0305	123.0440	122	C7H6O2	7.992	(-)121>101,97,89,78,77	-	REF[4]
5	Vanillic acid	4.75	167.0360	169.0495	168	C8H8O4	6.274	(-)167>148,123>108,95,79	256	REF[3]
6	4-Caffeoylquinic acid	4.96	353.0878	355.1024	354	C16H18O9	-0.015	(-)353>283,223,191,179>135>108,91	257	REF[5,6]
7	Diferulic acid	5.59	385.0943	387.1076	386	C20H18O8	3.660	(-)385>367,341>326,297,257>282	220, 279	REF[7,8]
Flavonoids										
8	Schaftoside	4.34	563.1415	565.1556	564	C26H28O14	1.512	(-)563>545, 473,443,413,383>353>325,297	215	REF[9]
9	Isoscoparin	4.34	461.1112	463.1238	462	C22H22O11	4.935	(-)461>417,341,>297,231>255,224	257	REF[10]
10	Baicalin	5.24	445.0796	447.0920	446	C21H18O11	4.394	(-)445>311,282, 269 >240,224>209,197,181,152	268	REF[11]
11	Isovitexin	5.53	431.0994	433.1130	432	C21H20O10	2.459	(-)431>413,388,341,327,309,283>281>253,238	220, 279	REF[12]
12	Chrysoeriol	5.76	299.0580	301.0709	300	C16H12O6	6.249	(-)299>283,256>239,226,213>198,183,170,155	223, 284	REF[12]
13	3,8-Dimethylherbacetin	6.43	329.0682	331.0810	330	C17H14O7	4.662	(-)329>314>299>271,227	219[8], 319	REF[13]
14	Calycosin	7.32	283.0627	285.0755	284	C16H12O5	5.240	(-)283>268,240>212>184	218, 257	REF[14]
Long-chain fatty acids										
15	Pinellic acid	6.54	329.2343	353.2292[f]	330	C18H34O5	4.327	(-)329>311,229,>211>183,167	226, 325	REF[15]
16	8,11-DiHODE	7.16	311.2228	313.2372	312	C18H32O4	0.184	(-)311>293>249,181>149	222, 277	REF[16]
Lysophospholipids										

17	LysoPG 14:0	7.89	455.2431	457.2569	456	C20H41O9P	3.355	(-)455>227>209,153>97,79	224, 279	REF[17]
18	LysoPC 14:0	8.01	512.3009[g]	468.3071	467	C22H46O7NP	3.139	(-)512>452>227>209	220	REF[16]
19	LysoPC 18:3	8.10	562.3167[g]	518.3230	517	C26H48O7NP	3.073	(-)562>502>277>259,233,179	222	REF[18]
20	LysoPE 18:2	8.46	476.2797	478.2920	477	C23H44NO7P	3.060	(-)476>279>261>243	230	REF[18]
21	LysoPC 18:2	8.51	564.3328[g]	520.3388	519	C26H50O7NP	3.913	(-)564>504>279>261	230	REF[15]
22	LysoPE 16:0	8.75	452.2799	454.2922	453	C21H44O7NP	3.510	(-)452>255>237>183<br>(+)454>436>393>239,154	224	REF[18]
23	LysoPC 16:0	8.81	540.3320[g]	496.3379	495	C24H50O7NP	2.477	(-)540>480>255>237<br>(+)496>478>419>283,163	224	REF[15]
24	LysoPE 18:1	8.96	478.2962	480.3079	479	C23H46O7NP	4.783	(-)478>281>263>245	225	REF[17]
25	LysoPC 18:1	9.04	566.3479[g]	544.3355[f]	521	C26H52O7NP	2.928	(-)566>507>281>263	225	REF[15]
26	LysoPE 18:0	9.58	480.3116	482.3232	481	C23H48O7NP	4.242	(-)480>283>265>121,83	226	REF[15]
27	LysoPC 18:0	9.71	568.3638[g]	524.3698	523	C26H54O7NP	3.234	(-)568>508,479>283>265<br>(+)525>506,184>447>311	225	REF[17]
Hydroquinones										
28	Auroglaucin	9.78	297.1501	299.1635	298	C19H22O3	1.454	(-)297>279,267,264,254,242,228>224>221,209	237, 279	GNPS[h], REF[19]
29	Dihydroauroglaucin	9.71	299.1659	301.1789	300	C19H24O3	1.979	(-)299>281,271,218>203,189,174,162>135	228, 267,311	GNPS, REF[19]
30	Tetrahydroauroglau-cin	10.04	301.1812	303.1951	302	C19H26O3	1.069	(-)301>283,273,217>204,189>175,161	228, 249, 295	GNPS
31	Flavoglaucin	10.43	303.1970	305.2111	304	C19H28O3	1.293	(-)303>275,204>219>191,148,135	226, 276	GNPS, REF[19]
Unknown										
32	N.I. (1)	6.28	313.0357	315.0501	314	C2H12O13N5	-0.586	(-)313>270>226>198,170	223, 282	-
33	N.I. (2)	7.85	415.1781	417.1901	416	C10H26O9N9	0.069	(-)415>397,309>294,264,252>236	225, 325	-
34	N.I. (3)	6.18	387.0728	389.0850	388	C5H18O15N5	0.354	(-)387>313,295>269>254,241,225	222, 280	-
35	N.I. (4)	8.24	331.1201	333.1328	332	C19H16O2N4	0.154	(-)331>287>259,243>215,187	227	-
36	N.I. (5)	8.50	388.2038	390.2172	389	C10H29O8N8	0.623	(-)388>319>235,207>190,166,139	227	-

37	N.I. (6)	9.72	523.1408	547.3546[f]	524	C17H26O14N5	0.863	(-)523>254>226>198	226	-
38	N.I. (7)	6.86	320.1409	322.1551	321	C5H21O8N8	-0.181	(-)320>251>208,182,167>139	219	-
39	N.I. (8)	5.81	401.0893	403.1022	402	C21H14O5N4	0.392	(-)401>358,327,309,283>281>253	-	-
40	N.I. (9)	6.51	322.1209	324.1708	323	C20H19O4	-0.488	(-)322>294,253>225>210,154	214, 280	-
41	N.I. (10)	5.87	327.0523	329.0655	328	C18H8O3N4	0.194	(-)327>283>268,240>212,184	215	-
42	N.I. (11)	8.17	567.1310	569.1431	568	C19H22O12N9	-0.908	(-)567>298>254>226	223, 274	-

[a] Tentative metabolites identified by comparing data with published literature and an in-house library
[a] Retention time (min)
[b] Molecular weight
[c] Mass
[d] Identifications
[e] Reference
[f] Adduct ion is sodium, $[M+Na]^+$
[g] Adduct ion is formic acid, $[M+COO]^-$
[h] Global natural products social molecular networking ; https://gnps.ucsd.edu/ProteoSAFe/static/gnps-splash.jsp

Table S2. List of significantly distinct metabolites from rice *koji* with different *Aspergillus spp*. during fermentation identified by GC-TOF-MS

No.	Ret(min)[a]	VIP[b]1	VIP2	Metabolites[c]	Unique Mass[d](m/z)	MS Fragment pattern(m/z)	TMS[e]	*p*-value	ID [f]
					Organic acid				
1	4.98	1.59	1.19	Lactic acid	117	73,147,117,75,66,148,191,59,74,190,133,	2	0.00000	Lib/STD
2	5.15	1.40	0.98	Acetic acid	177	147,66,55,177,133,148,58,52,93,76	2	0.00000	Lib/STD
3	7.00	1.15	1.06	Benzoic acid	179	179,105,77,135,51,180,136,106,50,194,78,76,181,	2	0.00000	Lib/STD
4	13.55	0.73	1.09	Ferulic acid	338	205,338,84,323,308,129,146,97	2	0.00000	Lib/STD
					Sugar & Sugar derivatives				
5	7.28	1.12	1.07	Glycerol	151	73,147,117,103,205,133,218,59,148,75,74,129,149	3	0.00000	Lib/STD
6	9.34	1.64	1.19	Erythritol	217	73,147,217,203,117,205,133,189,74,204	4	0.00000	Lib/STD
7	9.41	1.60	1.15	meso-Erythritol	307	147,73,103,217,117,189,205,191,129,13,116,148	4	0.00000	Lib/STD
8	10.68	0.80	1.16	Xylose	307	73,103,217,147,74,307,133,189,59,75	4	0.00000	Lib/STD
9	11.11	1.45	1.03	Adonitol	319	73,103,147,217,129,205,319,75,189,74	5	0.00000	Lib/STD
10	12.09	1.08	0.76	Mannose	319	157,73,189,319,163,247,205,244,160	5	0.00000	Lib/STD
11	12.45	1.46	1.04	Glucose	217	147,160,157,205,129,319,189	5	0.00000	Lib/STD
12	12.77	1.38	0.97	Gluconic acid	332	147,73,129,189,157,59,131,149	4	0.00000	Lib/STD
13	13.66	1.45	1.03	Myo-inositol	305	73,147,219,191,305,129,75,133,74	6	0.00000	Lib/STD
14	14.84	1.39	1.06	Glyceryl-glycoside	204	73,204,147,103,129,205,217,75,74,133,117,206	6	0.00000	Lib/STD
15	16.72	0.53	1.59	Sucrose	437	73,147,217,361,103,169,129,75,74,362	8	0.00000	Lib/STD
16	17.29	0.47	1.59	Maltose monohy-drate	204	73,204,75,217,129,147,103,74,117	8	0.00000	Lib/STD
					Fatty acid				
17	13.18	1.60	1.12	Palmitic acid	132	117,75,73,129,132,145,55,131,313,57	1	0.00000	Lib/STD
18	14.21	1.41	0.99	Linoleic acid	337	75,73,67,81,55,79,129,95,117,82	1	0.00000	Lib/STD
19	14.23	1.28	0.90	Oleic acid	339	75,73,117,129,55,145,67,96,81,84	1	0.00000	Lib/STD
20	14.35	1.35	0.95	Stearic acid	341	117,75,73,129,132,145,55,131,	1	0.00000	Lib/STD

[a] Retention time (min)
[b] variable importance projection (VIP)
[c] Tentative metabolites identified by mass spectrum consistent with those of standard compound, NIST, and in-house library and the different metabolites based on variable importance projection (VIP) analysis with a cutoff value of 1.0, and $p < 0.05$.
[d] *m*/*z* values are the selected ions for identification of derivatized metabolites
[e] Trimethylsilyl
[f] Identification: MS, mass fragment pattern; STD, standard compound

Figure S2. Correlation map of bioactivities (skin cell effect and antioxidant activity) and rice *koji* fermented with *Aspergillus cristatus* or *A. oryzae* metabolites according to Pearson's correlation coefficient. Each square indicates Pearson's correlation coefficient values (r). Red and blue a represent positive (0 < r < 1) and negative (-1 < r < 0) correlation, respectively.

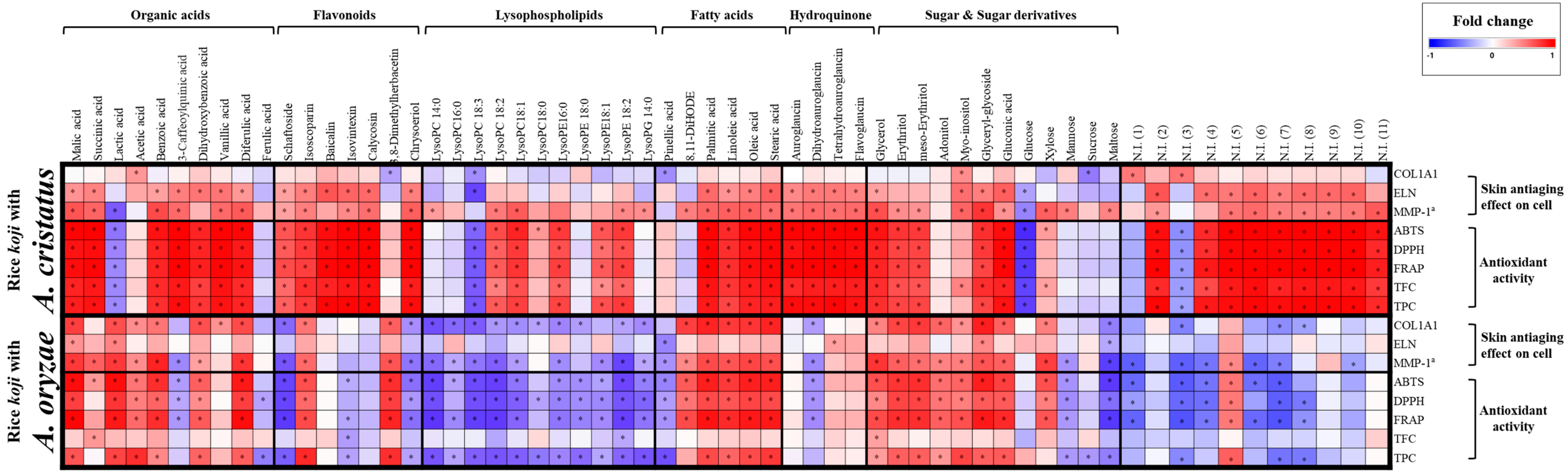

* $p < 0.05$

[a] Matrix metalloproteinase-1 (MMP-1); Negative correlation of MMP-1 was converted to positive correlation.

Supplementary References

1. Fernández-Fernández, R.; López-Martínez, J.C.; Romero-González, R.; Martínez-Vidal, J.L.; Alarcón Flores, M.I.; Garrido Frenich, A. Simple LC–MS Determination of Citric and Malic Acids in Fruits and Vegetables. *Chromatographia* **2010**, *72*, 55-62, doi:10.1365/s10337-010-1611-0.
2. Pawlak, M.; Klupczynska, A.; Kokot, Z.J.; Matysiak, J. Extending Metabolomic Studies of Apis mellifera Venom: LC-MS-Based Targeted Analysis of Organic Acids. *Toxins (Basel)* **2019**, *12*, doi:10.3390/toxins12010014.
3. Prabakaran, M.; Chung, I.-M.; Son, N.-Y.; Chi, H.-Y.; Kim, S.-Y.; Yang, Y.-J.; Kwon, C.; An, Y.-J.; Ahmad, A.; Kim, S.-H. Analysis of selected phenolic compounds in organic, pesticide-free, conventional rice (Oryza sativa L.) using LC-ESI-MS/MS. *Molecules* **2019**, *24*, 67.
4. Seraglio, S.K.T.; Valese, A.C.; Daguer, H.; Bergamo, G.; Azevedo, M.S.; Gonzaga, L.V.; Fett, R.; Costa, A.C.O. Development and validation of a LC-ESI-MS/MS method for the determination of phenolic compounds in honeydew honeys with the diluted-and-shoot approach. *Food Res Int* **2016**, *87*, 60-67, doi:10.1016/j.foodres.2016.06.019.
5. Li, Y.; Tang, W.; Chen, J.; Jia, R.; Ma, L.; Wang, S.; Wang, J.; Shen, X.; Chu, Z.; Zhu, C.; et al. Development of Marker-Free Transgenic Potato Tubers Enriched in Caffeoylquinic Acids and Flavonols. *J Agric Food Chem* **2016**, *64*, 2932-2940, doi:10.1021/acs.jafc.6b00270.
6. Kowalska, I.; Jedrejek, D.; Jonczyk, K.; Stochmal, A. UPLC–PDA–ESI–MS analysis and TLC–DPPH· activity of wheat varieties. *Acta Chromatographica* **2019**, *31*, 151-156.
7. Verardo, V.; Gómez-Caravaca, A.M.; Marconi, E.; Segura-Carretero, A.; Garrido-Frenich, A.; Fernández-Gutiérrez, A. Determination of lipophilic and hydrophilic bioactive compounds in raw and parboiled rice bran. *RSC advances* **2016**, *6*, 50786-50796.
8. Chiremba, C.; Taylor, J.R.N.; Rooney, L.W.; Beta, T. Phenolic acid content of sorghum and maize cultivars varying in hardness. *Food Chemistry* **2012**, *134*, 81-88, doi:https://doi.org/10.1016/j.foodchem.2012.02.067.
9. Colombo, R.; Yariwake, J.H.; McCullagh, M. Study of C-and O-glycosylflavones in sugarcane extracts using liquid chromatography: Exact mass measurement mass spectrometry. *Journal of the Brazilian Chemical Society* **2008**, *19*, 483-490.
10. Kim, B.; Woo, S.; Kim, M.-J.; Kwon, S.-W.; Lee, J.; Sung, S.H.; Koh, H.-J. Identification and quantification of flavonoids in yellow grain mutant of rice (Oryza sativa L.). *Food chemistry* **2018**, *241*, 154-162.
11. Fong, S.Y.K.; Wong, Y.C.; Zuo, Z. Development of a SPE-LC/MS/MS method for simultaneous quantification of baicalein, wogonin, oroxylin A and their glucuronides baicalin, wogonoside and oroxyloside in rats and its application to brain uptake and plasma pharmacokinetic studies. *Journal of pharmaceutical and biomedical analysis* **2014**, *97*, 9-23.
12. Galland, M.; Boutet-Mercey, S.; Lounifi, I.; Godin, B.; Balzergue, S.; Grandjean, O.; Morin, H.; Perreau, F.; Debeaujon, I.; Rajjou, L. Compartmentation and dynamics of flavone metabolism in dry and germinated rice seeds. *Plant and Cell Physiology* **2014**, *55*, 1646-1659.
13. Kong, C.; Xu, X.; Zhou, B.; Hu, F.; Zhang, C.; Zhang, M. Two compounds from allelopathic rice accession and their inhibitory activity on weeds and fungal pathogens. *Phytochemistry* **2004**, *65*, 1123-1128.
14. Kim, A.J.; Choi, J.N.; Kim, J.; Yeo, S.H.; Choi, J.H.; Lee, C.H. Metabolomics-based optimal koji fermentation for tyrosinase inhibition supplemented with Astragalus Radix. *Bioscience, biotechnology, and biochemistry* **2012**, 110171.
15. Kim, G.R.; Jung, E.S.; Lee, S.; Lim, S.-H.; Ha, S.-H.; Lee, C.H. Combined mass spectrometry-based metabolite profiling of different pigmented rice (Oryza sativa L.) seeds and correlation with antioxidant activities. *Molecules* **2014**, *19*, 15673-15686.
16. Son, S.Y.; Lee, S.; Singh, D.; Lee, N.-R.; Lee, D.-Y.; Lee, C.H. Comprehensive secondary metabolite profiling toward delineating the solid and submerged-state fermentation of Aspergillus oryzae KCCM 12698. *Frontiers in microbiology* **2018**, *9*, 1076.
17. Lim, D.K.; Mo, C.; Long, N.P.; Kim, G.; Kwon, S.W. Simultaneous profiling of lysoglycerophospholipids in rice (Oryza sativa L.) using direct infusion-tandem mass spectrometry with multiple reaction monitoring. *Journal of agricultural and food chemistry* **2017**, *65*, 2628-2634.
18. Lee, D.E.; Lee, S.; Jang, E.S.; Shin, H.W.; Moon, B.S.; Lee, C.H. Metabolomic profiles of Aspergillus oryzae and Bacillus amyloliquefaciens during rice koji fermentation. *Molecules* **2016**, *21*, 773.
19. Nies, J.; Ran, H.; Wohlgemuth, V.; Yin, W.-B.; Li, S.-M. Biosynthesis of the prenylated salicylaldehyde flavoglaucin requires temporary reduction to salicyl alcohol for decoration before reoxidation to the final product. *Organic letters* **2020**, *22*, 2256-2260.
